# Key Role of Human ABC Transporter ABCG2 in Photodynamic Therapy and Photodynamic Diagnosis

**DOI:** 10.1155/2010/587306

**Published:** 2010-07-08

**Authors:** Toshihisa Ishikawa, Hiroshi Nakagawa, Yuichiro Hagiya, Naosuke Nonoguchi, Shin-ichi Miyatake, Toshihiko Kuroiwa

**Affiliations:** ^1^Omics Science Center, RIKEN Yokohama Institute, 1-7-22 Suehiro-cho, Tsurumi-ku, Yokohama 230-0045, Japan; ^2^Graduate School of Bioscience and Biotechnology, Tokyo Institute of Technology, Yokohama 226-8501, Japan; ^3^Department of Neurosurgery, Osaka Medical College, Osaka 569-8686, Japan

## Abstract

Accumulating evidence indicates that ATP-binding cassette (ABC) transporter ABCG2 plays a key role in regulating the cellular accumulation of porphyrin derivatives in cancer cells and thereby affects the efficacy of photodynamic therapy and photodynamic diagnosis. The activity of porphyrin efflux can be affected by genetic polymorphisms in the *ABCG2* gene. On the other hand, Nrf2, an NF-E2-related transcription factor, has been shown to be involved in oxidative stress-mediated induction of the *ABCG2* gene. Since patients have demonstrated individual differences in their response to photodynamic therapy, transcriptional activation and/or genetic polymorphisms of the *ABCG2* gene in cancer cells may affect patients' responses to photodynamic therapy. Protein kinase inhibitors, including imatinib mesylate and gefitinib, are suggested to potentially enhance the efficacy of photodynamic therapy by blocking ABCG2-mediated porphyrin efflux from cancer cells. This review article provides an overview on the role of human ABC transporter ABCG2 in photodynamic therapy and photodynamic diagnosis.

## 1. Introduction

Photodynamic therapy (PDT) and photodynamic diagnosis are achieved by a photon-induced physicochemical reaction which is induced by excitation of photosensitizer exposed to light. In the 1960s Lipson and Baldes introduced a hematoporphyrin derivative (HpD), a product derived following by treatment of hematoporphyrin with a mixture of acetic and sulfuric acids and sodium hydroxide [1]. Their development of the hematoporphyrin derivative established the basis of today's PDT and photodynamic diagnosis [2–6]. PDT utilizes porphyrin derivatives to generate singlet oxygen (^1^O_2_) and other reactive oxygen species (ROS) that are potent in killing cancer cell *in vivo* [7]. The modern era of PDT was founded in the 1970s with the pioneering work of Dougherty and his coworkers who purified HpD later called Photofrin. In 1978, Dougherty et al. had carried out the first human trials of Photofrin on women with advanced breast cancer [8]. Photofrin is still the most widely used photosensitizer in clinical PDT. Recent studies of modern PDT began just two decades ago; therefore there are still unsolved problems. Nevertheless, PDT has many applications in a wide range of fields of both preclinical and clinical sciences.

In recent years, remarkable advances were made in photodynamic diagnosis technology that makes it easier to reliably achieve complete excision of malignant gliomas [9–11] and meningiomas [12]. The extent of tumor resection that should be undertaken in patients with glioblastoma multiforme remained controversial [13, 14]. Fluorescence-guided gross-total resection has been developed and it has prolonged the survival time of glioblastoma and meningioma patients [9–12, 15, 16]. Historically, two fluorescent agents, that is, fluorescein sodium and protoporphyrin IX (PpIX) induced by *δ*-Aminolevulinic acid (ALA) or its ester, have been used in glioma surgery. Because of its high tumor specificity and safety, ALA is particularly promising. It actively accumulates in the neoplasm and is converted to PpIX which is fluorescent. This phenomenon has been clinically applied to the detection of neoplasms in the brain and other organs, such as the bladder, skin, and bronchus. This technique is generally termed fluorescence diagnosis or photodynamic diagnosis. Fluorescence-guided resection may be beneficial for the removal of complicated or malignant tumors that have a high risk of recurrence. The most important point in fluorescence-guided microsurgery is the use of good equipment that can provide sufficient operative fields even under fluorescence mode.

## 2. Biosynthesis and Metabolism of Porphyrins and Heme

**Figure 1:**
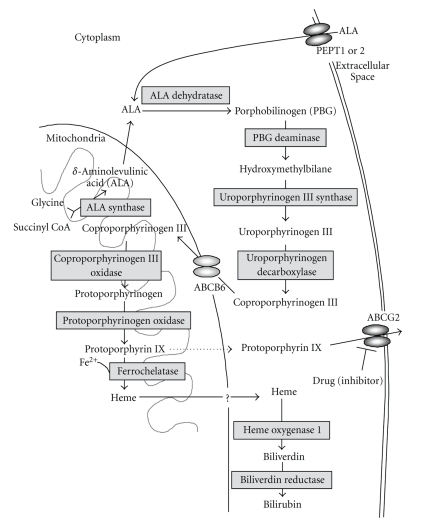
Schematic illustration of the biosynthesis and catabolism of heme. Rectangles indicate the enzymes involved in heme metabolism. Heme molecules are synthesized from glycine and succinyl Co-A via eight-stepped enzymatic reactions. ALA: *γ*-aminolevulinic acid; PBG: porphobilinogen. Heme, thus formed, is catabolized to biliverdin by the microsomal enzyme heme oxygenase 1. Biliverdin is subsequently metabolized to bilirubin by biliverdin reductase. ABC transporter ABCB6 is considered to be responsible for the import of coproporphyrinogen III into mitochondria, whereas ABCG2 transports porphyrins across the plasma membrane to maintain intracellular porphyrin homeostasis. ABCG2-mediated porphyrin transport can be inhibited by drugs, such as protein kinase inhibitors, as shown in Figure 5.

Hemes play critical roles in diverse biological processes, such as respiration and oxidative metabolism [17, 18]. Both heme biosynthesis and its intracellular concentration are tightly regulated. Heme molecules are formed via an eight-stepped pathway that is spatially shared between mitochondria and cytoplasmic compartments (Figure 1). At the first step, ALA is synthesized from glycine and succinyl Co-A in a reaction catalyzed by ALA-synthase and regulated by the intracellular-free heme pool. After ALA synthesis, a sequence of reactions occur leading to the production of various porphyrin compounds. Finally, as a result of ferrochelatase action, ferrous iron is incorporated into PpIX to form heme. Heme production as well as the synthesis of its two immediate precursors (PpIX and protoporphyrinogen) occurs in the mitochondria. ABCB6, one of human ABC transporters, reportedly transports coproporphyrinogen III from the cytoplasm to the mitochondria [19], whereas another ABC transporter ABCG2 is responsible for the cellular homeostasis of porphyrins and their related compounds [20]. Disturbances in cellular heme biosynthesis or metabolism are associated with several types of porphyria, which represent an elevation of toxic hemeprecursors, for example, protoporphyrins [21–23].

## 3. Enforced Biosynthesis of Protoporphyrin IX in Cancer Cells by ALA Administration

Exogenous ALA administration short-circuits the first step of porphyrin biosynthesis (Figure 1), where ALA is transported into cancer and normal cells by oligopeptide transporter 1 or 2 (PEPT1 or PEPT2). ALA induces the accumulation of detectable amounts of PpIX in certain types of cells, including cancer cells, making them photosensitive [24]. ALA-induced endogenous PpIX accumulation thus constitutes a photosentization process in which the selectivity of neoplastic cells in synthesizing and/or accumulating PpIX may be exploited to enhance the efficacy of PDT and photodynamic diagnosis.

PpIX has many other advantages. It is an essentially monomeric compound with a high fluorescence yield [25] and photosensitizing capability due to its good singlet oxygen quantum efficiency [26, 27] and is rapidly metabolized *in vivo* [28]. Animal and human studies have shown that ALA induces PpIX clearance from the skin within 24 hours after systemic, topical, or intradermal administration [24], whereas hematoporphyrin derivatives cause prolonged skin photosensitivity (1 to 2 months).

Not all cell lines can synthesize PpIX *in vitro* after ALA incubation in order to provide the reproducible *in vitro* assays required for *in vivo* studies of ALA-induced PDT. HepG2, a human hepatocarcinoma cell line, has been enzymatically well characterized to synthesize PpIX endogenously from exogenous ALA [29].

## 4. Photodynamic Diagnosis and Fluorescence-Guided Microsurgery

In photodynamic diagnosis and fluorescence-guided neurosurgery [9, 10, 12, 15, 16], ALA is used for intraoperative labeling of the border regions of malignant gliomas infiltrated by alive clonogenic tumor cells and is helpful in precise resection of those regions. ALA is converted to PpIX in the body and emits red fluorescence, with the excitation of blue-violet light. As PpIX preferentially accumulates in the tumor tissue in comparison with normal tissue, this red fluorescence becomes a good hallmark for discrimination between normal and tumor tissues, especially in malignant gliomas, which have infiltrative characteristics. Approximately 80% to 90% of the malignant gliomas show this red fluorescence in surgery, while only a limited number of metastatic brain tumor cases do not. In the surgery for metastatic brain tumor and lesionectomy for radiation necrosis and neurodegenerative disease, white matter around the lesion shows weak and vague fluorescence, which also provides us with a hallmark in the surgery. Additionally, in meningioma, some tumors showed the red fluorescence, which is especially helpful in the removal of the infiltrative portion in the bone and normal parenchyma [12]. Clinical data indicate that ALA-photodynamic diagnosis-assisted resection of malignant gliomas may result in statistically significant prolongation of postoperative survival [15, 16]. Ongoing research concentrates also on the use of ALA for a selective elimination of glioma cells *in situ* and on lipophilic ALA derivatives with more favorable pharmacokinetic properties.

**Figure 2:**
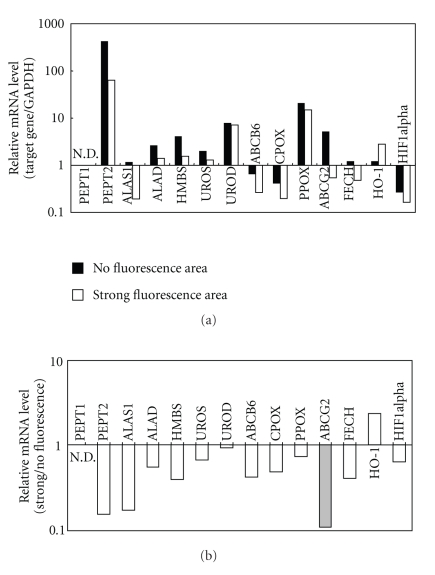
The mRNA levels of PEPT1, PEPT2, ALAS1, ALAD, HMBS, UROS, UROD, ABCB6 CPOX, PPOX, ABCG2, FECH, HO-1, and HIF1alpha in malignant glioma cells in the brain tumor and in the surrounding normal cells. Total RNA was extracted from strong fluorescence-emitting areas (brain tumor) as well as from the surrounding area (mostly normal tissue) without porphyrin fluorescence. The first strand cDNA was prepared from the extracted total RNA in a reverse transcriptase (RT) reaction. (a) The mRNA levels of the genes involved in the heme synthesis and metabolism reactions were detected by quantitative PCR and normalized to the mRNA level of GAPDH. (b) The relative mRNA levels of those genes in both areas were compared, where the relative mRNA level in the strong fluorescence-emitting area (brain tumor) was divided by that in nonfluorescence area (mostly normal tissue).

There is a question still remaining unanswered, namely: Why does PpIX accumulate in the tumor tissue more preferentially than in normal tissue? In order to answer for this question, we analyzed the expression levels of major enzymes and transporters involved in biosynthesis and metabolism of porphyrin. To determine mRNA levels of those enzymes and transporters, we design quantitative PCR primers (Table 1) and then compared their expression profiles between glioma and normal tissues (Figure 2(a)). The mRNA level of ABC transporter ABCG2 was found to be significantly lower in malignant glioma cells in the brain tumor that exhibited strong fluorescence of PpIX after ALA treatment (Figure 2(b)), whereas the surrounding normal cells emitted weak and vague fluorescence. These results suggest that ABCG2, a porphyrin efflux pump, is downregulated in tumors and thereby PpIX is facilitated to accumulate in cancer cells.

## 5. Human ABC Transporter ABCG2 in Cancer Chemotherapy and PDT

Human ABC transporter ABCG2, originally named Breast Cancer Resistant Protein (BCRP), was first discovered in doxorubicin-resistant breast cancer cells [30]. Since the same transporter has also been found in the human placenta [31] as well as in drug-resistant cancer cells selected in mitoxantrone [32], the transporter was also called ABCP or MXR1. The *ABCG2* gene is located on chromosome 4q22 and spans over 66 kb, comprising 16 exons and 15 introns. ABCG2 is classified in the G-subfamily of human ABC transporter genes according to the designated international nomenclature. Compared with the molecular structures of the well-known multidrug resistance transporters ABCB1 (P-gp/MDR1) and ABCC1 (MRP1), ABCG2 is a so-called “half ABC transporter” bearing six transmembrane domains and one ATP-binding cassette. Human ABCG2 has recently been shown to exist in the plasma membrane as a homodimer bound through disulfide-bonded cysteine residues [16, 33, 34]. Treatment with mercaptoethanol reduced the apparent molecular weight of ABCG2 from 140,000 to 70,000. Based on the cDNA sequence, a total of eleven cysteine residues exist in the ABCG2 protein. Among them, three cysteine residues in the extra-cellular loop of ABCG2 play pivotal roles in homodimer formation or protein expression levels. While Cys603 is involved in homodimer formation, Cys592 and Cys608 appear to be even more important for the formation of an intramolecular disulfide bond that greatly affects the protein stability as well as plasma membrane targeting of the ABCG2 protein [34, 35]. Recent studies have demonstrated that the *N*-linked glycan bound to Asn596 is important for stabilizing nascent ABCG2 proteins by facilitating homodimers in the endoplasmic reticulum (ER) [36, 37].

ABCG2 is endogenously expressed in placental trophoblast cells, in the epithelium of the small intestine and liver canalicular membrane, as well as in ducts and lobules of the breast. In particular, the high levels of ABCG2 expression in trophoblast cells suggest that the pump is responsible either for transporting compounds into the fetal blood supply or for removing toxic metabolites. The apical localization in the epithelium of the small intestine and colon indicates a possible role of ABCG2 in regulating the uptake of *p.o*. administered drugs [38]. It has recently been reported that gefitinib (iressa) enhanced the oral availability of irinotecan in mice [39], suggestive of the potential inhibition of mouse Abcg2 by gefitinib in the small intestine. On the other hand, ABCG2-knockout mice are extremely sensitive to the dietary chlorophyll-breakdown product pheophorbide a, which suggests that ABCG2 expressed in the small intestine plays a critical role in reducing the risk for developing diet-dependent phototoxicity and protoporphyria [40]. Importantly, ABCG2 has a high affinity to porphyrins, for example, hematoporphyrin and pheophorbide a [41]. ABCG2 transported protoporphyrin, hematoporphyrin, and pheophorbide a in an ATP-dependent manner [41–43].

## 6. Nrf2-Mediated Induction of ABCG2 and HO-1 by Photoactivation of Porphyrins

Recent evidence indicates that PDT can kill cancer cells directly by the efficient induction of apoptotic as well as nonapoptotic cell death pathways [7]. The identification of the molecular effectors regulating the cross-talk between cell death and cell protection pathways is an area of intense interest in the study of photokilling in cancer cells. 

**Figure 3:**
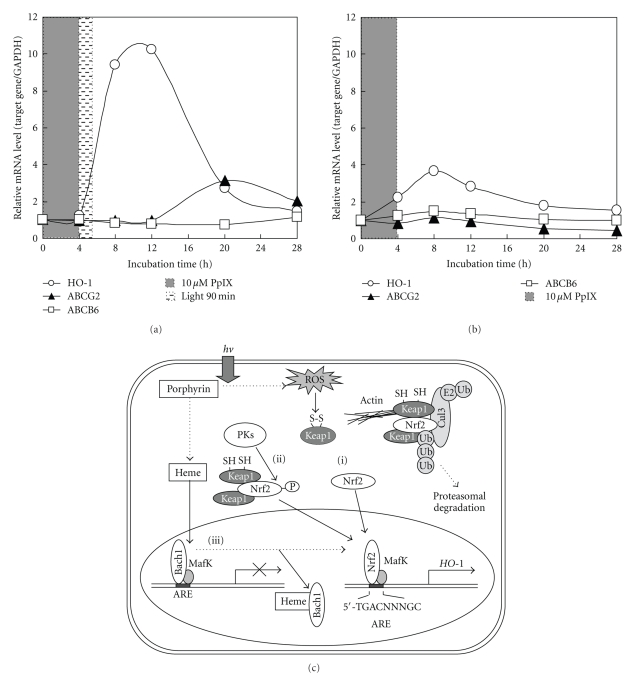
(a) Time courses of changes in the mRNA levels of heme oxygenase-1 (HO-1), ABCG2, and ABCB6 in HepG2 cells incubated with protoporphyrin IX (PpIX) and exposed to visible light. In dark conditions, HepG2 cells (1 × 10^6^ cells) were first incubated with 10 *μ*M PpIX in Dulbecco's modified Eagle's medium at 37°C under 5% CO_2_ gas for 4 hours. Thereafter, the incubation medium was replaced with fresh incubation medium. Cells were then exposed to visible light for 90 min, where cell culture dishes containing HepG2 cells were placed on a light viewer (Hakuba Model 5700) in an incubation chamber (37°C, 5% CO_2_). Cells were subsequently incubated in the dark at 37°C under 5% CO_2_ gas for 22.5 hours. The whole incubation was performed for a total of 28 hours, as indicated in the figure. (b) As the control experiments without light exposure, HepG2 cells (1 × 10^6^ cells) were incubated with 10 *μ*M PpIX in the same way as described above. PCR primers to quantitatively measure mRNA levels of HO-1, ABCG2, ABCB6, and GAPDH are described in [44]. (c) Schematic illustration for the activation of Nrf2 via three different pathways ((i), (ii), and (iii)). Pathway (i), under homeostatic conditions, Nrf2 is sequestered in the cytoplasm by the Keap1-Cul3 complex and rapidly degraded in a ubiquitin-proteasome-dependent manner. After an oxidative challenge (e.g., ^1^O_2_), oxidation of two reactive cysteine residues of Keap1 inhibits the ubiquitination reaction of Nrf2 mediated by the Keap1-Cul3 complex, which results in both cytoplasmic accumulation and nuclear translocation of Nrf2. Pathway (ii), activation of Nrf2 is mediated by protein kinases (PKs), such as p38^MAPK^, PI3K, PERK, and PKC. Pathway (iii), under normal conditions, the chromatin structure of HO-1 is in a preactivation state, but transcription is repressed by Bach1. Heme binds to Bach1, inhibiting its DNA binding activity and inducing its nuclear export. In the nuclei, the activated Nrf2 dimerizes with small Maf nuclear protein for effective binding to the ARE consensus sequence in the promoter region of the *HO-1* gene.

ABCG2 and heme oxygenase-1 (HO-1) are induced in HepG2 cells by photoactivation of porphyrins and display different induction patterns (Figures 3(a) and 3(b)). The induction of HO-1 was rapid and transient, whereas that of ABCG2 was relatively slow. Nevertheless, Nrf2-specific siRNA treatments caused significant impairments in the induction of both ABCG2 and HO-1 after the photoactivation of porphyrins [44], suggesting that Nrf2 is a common regulator for the transcriptional activation of *ABCG2* and *HO-1* genes.

Nrf2 is a basic region-leucine zipper- (bZip-) type transcription factor and plays a critical role in transcriptional upregulation of many target genes, including those for metabolizing enzymes and transporters essential for cellular defense in response to oxidative and/or electrophilic stress [45, 46]. Nrf2 targets the ARE containing the consensus sequence of 5*'*-A/GTGACNNNGC) [45]. Keap1, on the other hand, is known to be a negative regulator of Nrf2 by retrieving it in the cytoplasm (Figure 3(c)). Oxidative stress and/or electrophilic attack lead to the dissociation of Nrf2 from Keap1 and thereby activate Nrf2 for transcriptional regulation of ARE-dependent genes. Indeed, many genes encoding detoxifying and antioxidant enzymes were found to be regulated by Nrf2 [46, 47].

We have recently reported the potential role of the Nrf2/Keap1 mechanism in the induction of human ABC transporters as well as HO-1 in HepG2 cells under oxidative stress [44, 48]. The siRNA-mediated knockdown of Nrf2 or Keap1 revealed that the induction of ABCG2 and HO-1 under oxidative stress involves an Nrf2-dependent mechanism. However, the time course of ABCG2 induction is different from that of HO-1. While the Nrf2/Keap1 mechanism may be involved in the oxidative stress-mediated ABCG2 induction, the induction mechanism may be indirect. Thus, we consider an alternative mechanism of so-called “*trans-*activation” for the induction of the *ABCG2* gene [48].

The activation and nuclear translocation processes of Nrf2 in HO-1 induction seem to be more complex than previously expected. As shown in Figure 3(c), at least three distinct pathways may be involved in the activation of the Nrf2 protein leading to HO-1 induction: oxidation of critical cysteinyl residues of the Keap1 protein and concomitant inhibition of ubiquitination activity of Keap1 (Pathway i); phosphorylation of the Nrf2 protein via protein kinases, such as p38^MAPK^, PI3K, PKC, and PERK (Pathway ii); and direct binding of heme to Bach1 and facilitation of Nrf2/small Maf heterodimer formation (Pathway iii).

The HO-1 induction is dependent on transcription and *de novo* protein synthesis, and it is preceded by the nuclear accumulation of the Nrf2 transcription factor. In pathway (i), Nrf2 is repressed under quiescent conditions, where Keap1 and Cul3 constitute a unique ubiquitin E3 ligase that leads to the degradation of Nrf2. Upon exposure to oxidants/electrophiles, the enzymatic activity of this ligase complex is inhibited and the complex fails to degrade Nrf2, resulting in the transcriptional activation of Nrf2 target genes [47, 49]. Cys151 of Keap1 reportedly plays an important role to facilitate Nrf2 activation in response to oxidants/electrophiles [49].

Regarding pathway (ii), Martin et al. [50] reported that HO-1 expression is regulated through the PI3K/Akt pathway and the Nrf2 transcription factor in response to the antioxidant phytochemical carnosol. Kocanova et al. [51] more recently characterized the signaling pathways and the mechanisms leading to the upregulation of HO-1 in cancer cells subjected to hypericin-based PDT. They have shown that HO-1 induction mechanisms involve the p38^MAPK^ and PI3K signaling cascade. Besides p38^MAPK^ and PI3K, the activation of Nrf2 is mediated by other protein kinases, such as PERK and PKC, being dependent on cell types [52–57].

In pathway (iii), heme regulates the dynamic exchange of Bach1 and Nrf2 in the Maf transcription factor network. Igarashi and his colleagues proposed this direct interaction model [58–61]. The transcription repressor Bach1 is a sensor and effector of heme that regulates the expression of *HO-1* and *globin *genes [62–65]. Under normal conditions, the chromatin structure of HO-1 is in a preactivation state, but transcription is repressed by Bach1. Heme binds to Bach1, inhibiting its DNA binding activity [66] and inducing its nuclear export [67]. Furthermore, heme induces ubiquitination and degradation of the transcription factor Bach1 [68]. As a consequence, heme induces the switching of Nrf2/small Maf hetero-dimers, resulting in HO-1 expression [69].

## 7. Clinical Implications of Nrf2-Mediated Induction of HO-1

The induction of HO-1 after photoactivation of PpIX or Pheo a is most probably mediated by pathway (i), whereby ^1^O_2_ generated from the photooxidative reaction could oxidize the critical cysteine residues of Keap1 and thereby increase the nuclear availability of the Nrf2 pool. HO-1 catalyzes the oxidation of heme to biologically active products: carbon monoxide (CO), biliverdin, and ferrous iron. It participates in maintaining cellular homeostasis and plays an important protective role in the tissues by reducing oxidative injury, attenuating the inflammatory response, inhibiting cell apoptosis, and regulating cell proliferation. Induction of HO-1 by hemin prior to irradiation is cytoprotective [51]. A growing body of evidence indicates that HO-1 activation plays a pivotal role in the progression of tumors. HO-1 is very often upregulated in tumor tissues, and its expression is further increased in response to therapies. Many studies have convincingly shown that upregulation of HO-1 significantly improves survival of hepatoma, melanoma, thyroid carcinoma, chronic myelogenous leukemia, gastric cancer, and colon cancer cell lines [70]. Increased transcription and translation of HO-1 was observed in Chinese hamster fibroblasts following photodynamic stress or photofrin II incubation [71]. Nowis et al. recently demonstrated that HO-1 protected tumor cells against PDT-mediated cytotoxicity [72]. On the other hand, HO-1 inhibitors or targeted knockdown of HO-1 expression made the cultured cell lines much more sensitive to anticancer therapy. Accordingly, inhibition of HO-1 can be suggested as a potential therapeutic approach sensitizing tumors to radiation, chemotherapy, or PDT [70].

## 8. Pharmacogenomics of ABCG2 and Potential Impact on PDT

**Figure 4:**
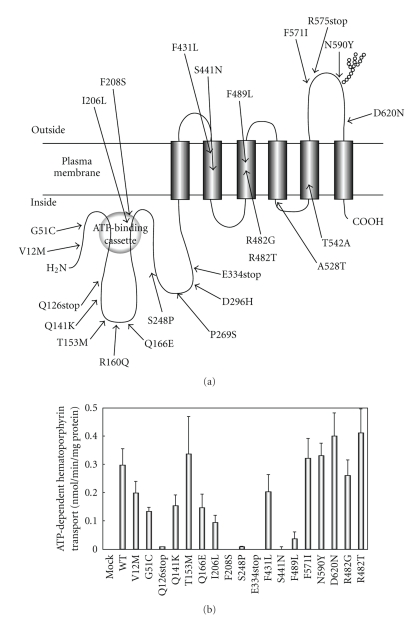
(a) Schematic illustration of human ABCG2 and its nonsynonymous polymorphisms. SNP data on the polymorphisms of ABCG2 were obtained from the NCBI dbSNP database and recent publications. The variants R482G and R482T are acquired mutations. (b) ATP-dependent transport of hematoporphyrin by ABCG2 and its variants. Plasma membrane vesicles expressing ABCG2 variants (50 *μ*g of protein) were incubated with 20 *μ*M hematoporphyrin in the presence or absence of 1 mM ATP in the standard incubation medium at 37°C for 10 minutes. The ATP-dependent transport of hematoporphyrin is normalized for the amount of ABCG2 protein. Data are from [41].

Single nucleotide polymorphisms (SNPs) of the *ABCG2* gene have been suggested to be a significant factor affecting patients' responses to medication and/or the risk of diseases [73–77]. Sequencing of the *ABCG2* gene from human samples has revealed over 80 different, naturally occurring sequence variations [75–77]. Among them, a total of 17 nonsynonymous polymorphisms have been reported for the ABCG2 gene [41, 74, 78] (Figure 4(a)).

The most extensively studied among those SNPs with potential clinical relevance is 421 C > A resulting in a glutamine to lysine substitution (Q141K) in the ABCG2 protein. The Q141K SNP has been identified with varying frequencies in different ethnic groups and was found to be the most relevant in Japanese and Chinese populations (approximately 30% in the allele frequency). Low allele frequencies (0% to 35%) are found in the Africans North of the Sahara, sub-Saharan Africans, and African-American subjects [74].

Q141K has been associated with lower levels of protein expression and impaired transport *in vitro* [79–84]. The polymorphism has been studied *in vivo*; patients carrying the SNP were found to have elevated plasma levels of gefitinib, diflomotecan, and increased bioavailability of oral topotecan [85–87]. Furthermore, the Q141K SNP was reportedly associated with a higher incidence of diarrhea in nonsmall cell lung cancer patients treated with gefitinib [88]. It has been demonstrated that the reduced expression levels of the Q141K variant may be due to its ubiquitin-mediated proteasomal degradation [89].

The apparent Km value of ABCG2 WT toward hematoporphyrin was estimated to be 17.8 *μ*M [41]. To clarify the possible physiological or pathological relevance of ABCG2 polymorphisms, we have functionally validated polymorphisms of ABCG2 [19, 41, 42, 78]. Based on the currently available data on SNPs and acquired mutations, we have created a total of 18 variant forms of ABCG2 (V12M, G51C, Q126stop, Q141K, T153M, Q166E, I206L, F208S, S248P, E334stop, F431L, S441N, R482G, R482T, F489L, F571I, N590Y, and D620N) by site-directed mutagenesis and expressed them in insect cells [41, 90]. The variants Q126stop, F208S, S248P, E334stop, and S441N are defective in the transport of hematoporphyrin (Figure 4(b)). The F489L variant showed impaired transport activity (Figure 4(b)). Flp-In-293 cells expressing the F208S, S248P, S441N, and F489L variants were sensitive to light when cells were treated with pheophorbide a. Thus, it is likely that humans with these alleles may be more susceptible to porphyrin-induced phototoxicity.

## 9. Interaction of ABCG2 with Protein Kinase and CDK Inhibitors

Protein kinases are potential drug targets for the treatment of a variety of diseases, including cancer [91]. In particular, specific tyrosine kinase inhibitors are rapidly being developed as new drugs for the inhibition of malignant cell growth and metastasis. Most of these newly developed tyrosine kinase inhibitors are hydrophobic and thus rapidly penetrate the cell membrane to reach intracellular targets.

The human genome encodes more than 500 protein kinases, and this protein kinase family has been the subject of intensive research for the development of novel anticancer drugs [92]. Gefitinib and imatinib are new anticancer drugs that have been developed as inhibitors for EGFR tyrosine kinase and BCR/ABL kinase, respectively. Recently, another class family of protein kinases has been attracting particular attention, namely, the cyclin-dependent kinases (CDKs), which regulate critical processes of cell cycle progression and gene transcription essential for cancer cell survival [93]. In cancer, CDKs are deregulated in different ways, such as by the overexpression of cyclin E [94] and loss of p16^INK4A^, a CDK inhibitor [95]. Thus, small-molecule chemicals that specifically regulate or inhibit CDKs are of great interest in drug discovery and development for cancer chemotherapy.

The QSAR analysis revealed that a structure having one amine bonded to one carbon of a heterocyclic ring is an important component for interaction with the ABCG2 protein [96]. In addition, fused heterocyclic ring(s) and two substituents on a carbocyclic ring of the fused heterocyclic ring(s) are also important chemical moieties for the interaction with ABCG2 [96]. Interestingly, many protein kinase inhibitors carry such structural components within their molecules. Based on the QSAR analysis, we hypothesized that those CDK inhibitors would interact with the ABCG2 protein [37].

## 10. Photosensitivity Evoked by Inhibition of ABCG2-Mediated Porphyrin Transport

Clinical photosensitizers, such as protoporphyrin, 2-(1-hexyloxethyl)-2-devinyl pyropheophorbide a (photochlor), and benzoporphyrin derivative monoacid ring A (Verteporfin), were transported out of cells by ABCG2, whereas this effect was abrogated by the coadministration of imatinib mesylate [97]. By increasing intracellular photosensitizer levels in ABCG2-positive tumors, imatinib mesylate or other ABCG2 transport inhibitors may enhance the efficacy and selectivity of clinical PDT [98]. In this regard, we have reported that cellular phototoxicity was evoked through the inhibition of human ABC transporter ABCG2 by imatinib [42] and cyclin-dependent kinase (CDK) inhibitors [43].

**Figure 5:**
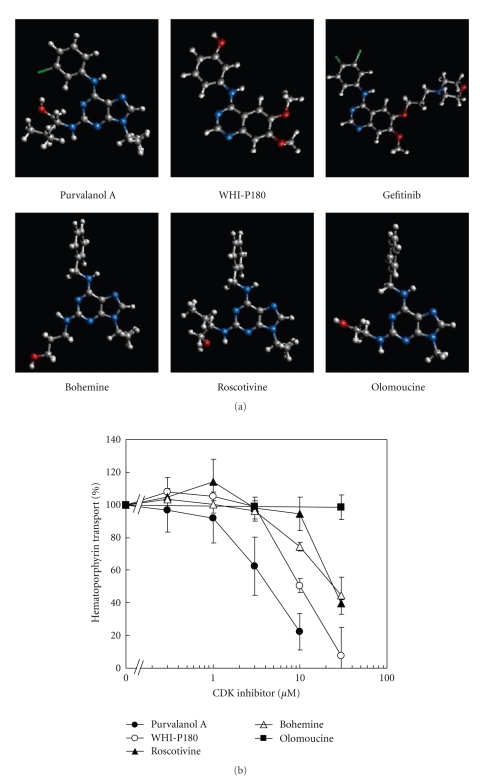
(a) Chemical structures of CDK inhibitors: purvalanol A, WHI-P180, gefitinib, bohemine, roscovitine, and olomoucine. (b) Inhibition of ABCG2-mediated hematoporphyrin transport by purvalanol A, WHI-P180, roscovitine, bohemine, or olomoucine. ABCG2-expressing plasma membrane vesicles (20 *μ*g of protein) were incubated with 20 *μ*M hematoporphyrin in the presence of purvalanol A, WHI-P180, roscovitine, bohemine, or olomoucine (final concentration: 0, 0.3, 1, 3, 10, or 30 *μ*M) in the standard incubation medium (0.25 M sucrose and 10 mM Tris/HEPES, pH 7.4, 1 mM ATP, 10 mM creatine phosphate, 100 *μ*g/mL of creatine kinase, 10 mM MgCl_2_) at 37°C for 10 min. Hematoporphyrin transported into membrane vesicles was detected [43]. Data are expressed as mean values ± SD (*n* = 5).

To gain further insights into drug-ABCG2 interactions, the three-dimensional (3D) structures of those CDK inhibitors (Figure 5(a)) were generated by *ab initio* MO calculation [43]. Purvalanol A and WHI-P180 have a planar structure, whereas bohemine, roscovitine, and olomoucine do not. In the latter CDK inhibitors, the aromatic ring is orthogonal to the purine ring [43]. Among the CDK inhibitors tested (i.e., purvalanol A, WHI-P180, bohemine, roscovitine, and olomoucine), purvalanol A was found to be the most potent inhibitor for ABCG2-mediated hematoporphyrin transport (Figure 5(b)). Accordingly, it evoked the photosensitivity of ABCG2-expressing Flp-In-293 cells treated with pheophorbide a [43]. Thus, it is suggested that the planar structure is an important factor for interactions with the active site of ABCG2. Taken together, ABCG2 is one of the critical factors that affect the efficacy of PDT as well as chemotherapy of human cancer.

## 11. Concluding Remarks

ABCG2 was discovered a decade ago and has been studied in laboratories around the world, yielding a wealth of knowledge akin to that gathered for ABCB1 (P-glycoprotein/MDR1) and ABCC1 (MRP1). Chemotherapy and PDT are effective treatment options for human cancer. However, individual differences among patients were also noticed in their responses to those therapies. Gupta et al. demonstrated interesting data that ABCG2 mRNA was present in normal colorectal tissue but showed a 6-fold decrease in colorectal cancer [98]. The downregulation of ABCG2 mRNA and protein was also evident in cervical cancer. Indeed, we have also found that ABCG2 expression was downregulated in malignant glioma of human brain tumor (Figure 2). These observations indicate that cancer-associated downregulation of ABCG2 is likely to be a common phenomenon in several tumors and that the accumulation of clinical photosensitizers in cancerous tissues may be due, in part, to the reduced expression levels of ABCG2 in cancer cells. On the other hand, induction of ABCG2 and HO-1 is considered as critical factors for PDT, and they can be readily induced by photo-oxidative stress to protect cancer cells. Thus, investigations of molecular mechanisms underlying the induction of those genes would be of importance to create new strategies for individualized PDT.

## Figures and Tables

**Table 1:** PCR primers to quantitatively measure the mRNA levels of PEPT1, PEPT2, ALAS1, ALAD, HMBS, UROS, UROD, ABCB6 CPOX, PPOX, ABCG2, FECH, HO-1, and HIF1alpha.

Gene	F/R	Primer sequence	Position	Tm
PEPT1	Forward	CCCTGAAGTGAAGGTGTTTGAAGATA	1772–1797	60.6
NM_005073	Reverse	GAATTGGCCCCTGACATGAA	2168–2188	59.3
PEPT2	Forward	CTACCACAATATGCCCTGGTTACA	1894–1917	58.8
NM_001145998	Reverse	GCCACTGAACTGTGCCACAA	2045–2064	59.3
ALAS1	Forward	GGATTCGAAACAGCCGAGTG	1371–1390	59.3
NM 000688	Reverse	GAAGGTGATTGCTCCAAACTCAT	1542–1564	58.3
ALAD	Forward	CCTCGGTTCCAACCAACTGAT	157–177	59.8
NM_000031	Reverse	GATAGGCTGTATGTCATCAGGAACA	319–343	58.2
HMBS	Forward	CAAGGACCAGGACATCTTGGAT	835–856	58.9
NM_000190	Reverse	CCAGACTCCTCCAGTCAGGTACA	984–1006	59.2
UROS	Forward	TCAGCACTGCCTCTTCTATTTCC	668–691	58.7
NM_000375	Reverse	CTGGGTGTGCAACTGTCTGATAC	761–789	58.3
UROD	Forward	CGGGAGTGTGTGGGGAA	982–998	57.2
NM_000374	Reverse	AAGCAGACGTGAGTGTTTATGCA	1178–1200	58.6
ABCB6	Forward	CAGAAGGGCCGTATTGAGTTTG	2033–2054	59.6
NM_005689	Reverse	ATTGTCGGCGATGGTGTCA	2308–2326	59.5
CPOX	Forward	GGCGGAGATGTTGCCTAAGAC	401–421	59.7
NM_000097	Reverse	AATGCTCACCCCAGCCTTTT	709–728	59.5
PPOX	Forward	CAGGAGTCCTGGGAATCGTGTA	1251–1272	59.9
NM_000309	Reverse	TGCCTAGCTGACTCTAGTTTTTGC	1509–1533	58.1
FECH	Forward	GGAAATCCATTGTTCTCTAAGGC	1252–1274	57.0
NM_001012515	Reverse	CTAAATAACACCCTCTCCACATCG	1462–1485	57.8
ABCG2	Forward	CTAAGCAGGGACGAACAATCATC	1188–1210	58.8
NM_004827	Reverse	TCCTGCTTGGAAGGCTCTATG	1447–1467	58.2
HIF-1alpha	Forward	GGCGCGAACGACAAGAA	420–436	58.0
NM 001530	Reverse	CAAAACCATCCAAGGCTTTCA	682–702	58.7
HO-1	Forward	GCTCAAAAAGATTGCCCAGAA	518–538	58.1
NM_002133	Reverse	TCACATGGCATAAAGCCCTACA	926–947	59.1

PEPT1: oligopeptide transporter 1; PEPT2: oligopeptide transporter 2; ALAS1: delta-aminolevulinate synthase 1; ALAD, delta-aminolevulinate dehydratase; HMBS: hydroxymethylbilane synthase; UROS: uroporphyrinogen III synthase; UROD: uroporphyrinogen decarboxylase; ABCB6: ABC transporter B6; CPOX: coproporphyrinogen oxidase; PPOX: protoporphyrinogen oxidase; FECH: ferrochelatase; ABCG2: ABC transporter G2 (BCRP); HIF-1alpha: hypoxia inducible factor-1 alpha subunit; HO-1: heme oxygenase-1. F/R: forward or reverse primers; Tm: melting temperature.
